# Importance of SLC26 Transmembrane Anion Exchangers in Sperm Post-testicular Maturation and Fertilization Potential

**DOI:** 10.3389/fcell.2019.00230

**Published:** 2019-10-18

**Authors:** Aminata Touré

**Affiliations:** INSERM U1016, Centre National de la Recherche Scientifique, UMR 8104, Institut Cochin, Université de Paris, Paris, France

**Keywords:** SLC26, CFTR, pH, sperm, epididymis, motility, capacitation, fertilization

## Abstract

In mammals, sperm cells produced within the testis are structurally differentiated but remain immotile and are unable to fertilize the oocyte unless they undergo a series of maturation events during their transit in the male and female genital tracts. This post-testicular functional maturation is known to rely on the micro-environment of both male and female genital tracts, and is tightly controlled by the pH of their luminal milieus. In particular, within the epididymis, the establishment of a low bicarbonate (HCO_3_^–^) concentration contributes to luminal acidification, which is necessary for sperm maturation and subsequent storage in a quiescent state. Following ejaculation, sperm is exposed to the basic pH of the female genital tract and bicarbonate (HCO_3_^–^), calcium (Ca^2+^), and chloride (Cl^–^) influxes induce biochemical and electrophysiological changes to the sperm cells (cytoplasmic alkalinization, increased cAMP concentration, and protein phosphorylation cascades), which are indispensable for the acquisition of fertilization potential, a process called capacitation. Solute carrier 26 (SLC26) members are conserved membranous proteins that mediate the transport of various anions across the plasma membrane of epithelial cells and constitute important regulators of pH and HCO_3_^–^ concentration. Most SLC26 members were shown to physically interact and cooperate with the cystic fibrosis transmembrane conductance regulator channel (CFTR) in various epithelia, mainly by stimulating its Cl^–^ channel activity. Among SLC26 members, the function of SLC26A3, A6, and A8 were particularly investigated in the male genital tract and the sperm cells. In this review, we will focus on SLC26s contributions to ionic- and pH-dependent processes during sperm post-testicular maturation. We will specify the current knowledge regarding their functions, based on data from the literature generated by means of *in vitro* and *in vivo* studies in knock-out mouse models together with genetic studies of infertile patients. We will also discuss the limits of those studies, the current research gaps and identify some key points for potential developments in this field.

## Introduction

**FIGURE 1:**
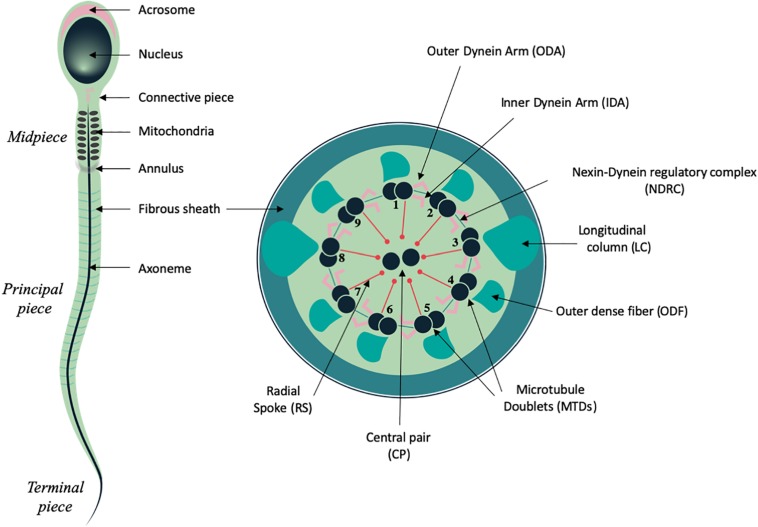
Schematic representation of mammalian spermatozoa and flagellum structure. **Left panel**, overall view of the spermatozoa showing the main head and flagellum structures and compartments. **Right panel**, cross section from the principal piece of the flagellum showing the organization of the axoneme: microtubule doublets (MTD), central pair (CP), radial spokes (RS), nexin dynein regulatory complex (NDRC), inner and outer dynein arms (IDA and ODA), together with some of the peri-axonemal structures: fibrous sheath (FS), outer dense fiber (ODF), and longitudinal columns (LC).

Spermatozoa constitute one of the most differentiated cell types of the body and are produced within the seminiferous tubules of the testis during spermatogenesis, a complex and tightly regulated process of nearly 2.5 months long in humans and 7 weeks in mice (Heller and Clermont, 1964; Clermont, 1972). At the end of this process, spermatozoa are fully differentiated at the morphological level, and comprise two main compartments: the head and the tail, each fulfilling specific functions that are essential for fertilization (Figure 1). The sperm head comprises the nucleus, in which the haploid paternal DNA is highly compacted through replacement of histones by protamines that mediate hyper condensation of the chromatin during spermiogenesis (Miller et al., 2010). In addition, the acrosome, a peculiar vesicle which derives from the Golgi, locates to the anterior half of the sperm head and forms a large cap containing various proteases and membrane receptor that are required to cross the cumulus cell layer and digest the zona pellucida that surround the oocyte (Foster and Gerton, 2016). The tail or flagellum is an organelle of 50 to 100 micrometer long in mammals, which sustains sperm motility and progression within the female genital tract and is thus also indispensable for fertilization. It is composed of an evolutionary conserved microtubule-based structure, called the axoneme, which is also shared with cilia, and contains nine microtubules doublets (MTD) organized around a central pair of microtubules (CP) (Inaba, 2007, 2011). Attached to the MTD, the Inner and Outer Dynein Arms (IDAs and ODAs), which constitute multiprotein complexes with ATPase activity, drive the sliding of the MTD, and orchestrate the sperm flagellum beating. In addition, MTD are connected to each other through the nexin-dynein regulatory complex (NDRC) and to the CP complex via the radial spokes (RS) (Inaba, 2007, 2011; Figure 1). This latter multi-protein complex ensures the stability of the axonemal structure and may also function as a scaffold for signaling molecules such as Calmodulin (CaM) and Protein kinase A (Yang et al., 2006).

In spermatozoa from primitive species such as fishes, the flagellum is similar to that of cilia and only comprises the microtubular axoneme surrounded by the plasma membrane. In mammals, the sperm cells harbor peri-axonemal structures that surround the microtubule-based cytoskeleton of the tail beneath the plasma membrane and are required for structural cohesion, energy regulation and cell signaling (Eddy et al., 2003; Eddy, 2007). The structure of mammalian sperm tail can be divided in several compartments, based on their content on peri-axonemal structures. Hence, the midpiece (MP) is characterized by the presence of the mitochondrial sheath (MS) and contains outer dense fibers (ODFs), which ensure elasticity and structural integrity (Lehti and Sironen, 2017). The principal piece (PP) is characterized by the fibrous sheath (FS), which comprises two longitudinal columns attached to doublets 3 and 8 that partially displace ODFs and are connected by semi-circular ribs. Proteins from the FS are stabilized by di-sulfide bonds, which suggest that the FS might strengthen the sperm structure and influence its flexibility (Eddy et al., 2003). The FS also behaves as a scaffold for proteins regulating sperm motility and functionality as it harbors glycolytic enzymes and signaling molecules, such as AKAP proteins and cAMP-dependent protein kinase (Eddy, 2007; Lehti and Sironen, 2017). Lastly, the terminal piece encloses the sperm tail and is only composed of the axoneme. In addition, two specific structures of the sperm tail are also distinguishable: the connecting piece, which anchors the tail to the sperm head (Inaba, 2007, 2011) and the annulus, a Septin-ring structure (also called Jensen’s ring), which locates at the boundary of the midpiece and the principal piece and acts as a diffusion barrier to ensure the correct localization of proteins along the different compartments of the sperm flagellum (Toure et al., 2011; Figure 1).

While spermatozoa released from the testicular seminiferous tubules are morphologically differentiated, they are immotile and unable to recognize and fertilize the oocyte. Importantly, sperm functionality will be conferred by a series of maturation events occurring during their transit through the male and female genital tracts (Fraser, 1992; Yeung and Cooper, 2003). Such post-testicular functional maturation is known to rely on the luminal milieus of the male and female genital tracts, which composition results from specific absorptive and secretory activities of epithelial cells that line the lumen of both tracts. In particular, sperm maturation is tightly controlled by the pH of the luminal milieu. In the first part of this review we will describe the current knowledge on (i) the cellular cross-talks and membrane transporters that are involved in the establishment of the acidic luminal milieu required for epididymal maturation, and (ii) the ionic fluxes and physiological changes occurring in the sperm cells upon capacitation, which are triggered by sperm exposition to high concentration of HCO_3_^–^ in the female genital tract. We will next focus on SLC26 proteins, a well-established transmembrane protein family involved in anionic transport and pH regulation in various epithelia. We will provide a comprehensive description of their contributions in the regulation of some hallmarks associated with capacitation and present recent data, which revealed their functions within the epididymal cells. Lastly, we will describe the human disorders related to SLC26 dysfunctions in the male reproductive organs. We will conclude the review by discussing some of the current research gaps in this field and presenting potential perspectives for future research.

## Sperm Epididymal Maturation

### Epididymis Structure and Function

**FIGURE 2 F2:**
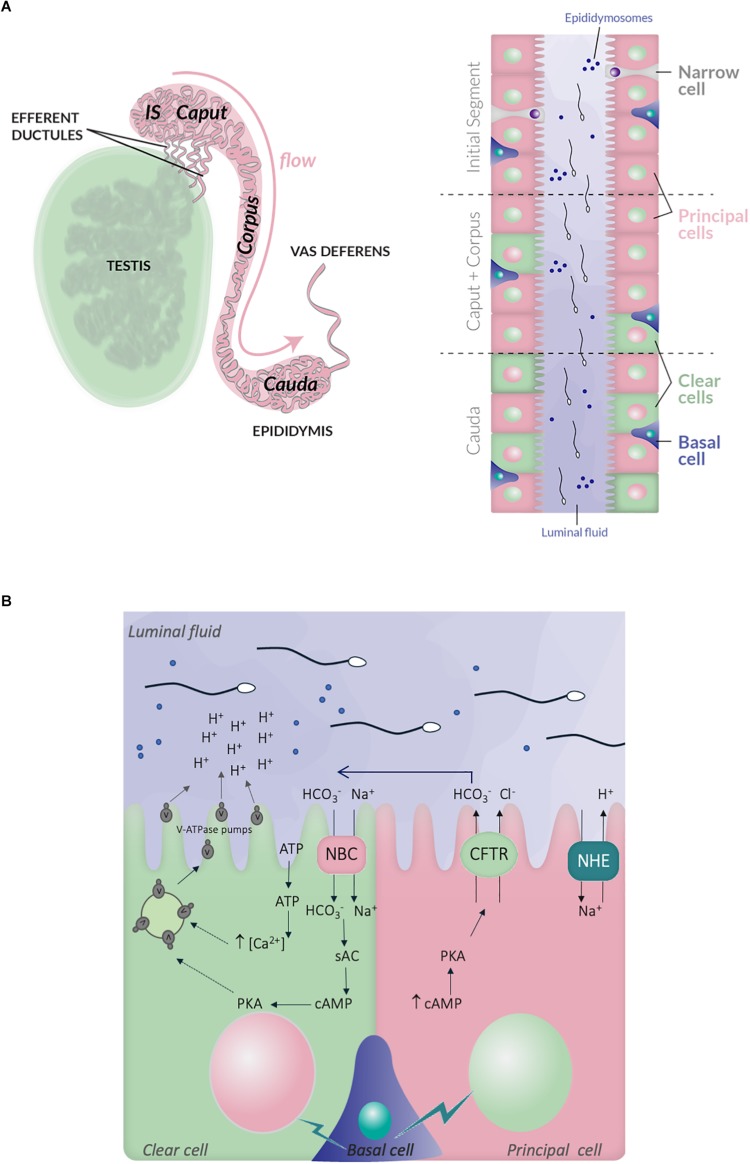
Schematic representation of the epididymis structure and ionic exchanges between epithelial cells, which control luminal acidification. **(A)** Left panel, the testis, efferent ductules and epididymis are schematized. The different regions within the epididymis, in mouse, are indicated: initial segment, caput, corpus and cauda, following the proximal-distal axis. Right panel, the distribution of the different epithelial cell-types within the epididymal tract is also illustrated: principal cells (PCs), clear cells (CCs), narrow cells (NCs), and basal cells; the luminal fluid shows epididymosomes, which are small vesicles transferring material from epithelia cells to the sperm cells. **(B)** Simplified representation of the main ionic fluxes and cross talks occuring between principal, clear, and basal cells. CCs expressed the V-ATPase pumps, which expression at the plasma membrane is induced by HCO_3_^–^ and c-AMP dependent pathway. The HCO_3_^–^ influx in CCs is mediated by the NBC sodium- HCO_3_^–^ transporter. ATP also induces intracellular rise of Ca^2+^, which increase V-ATPase translocation at the plasma membrane and proton secretion. PCs express the NHE3 sodium-proton antiporter, which contributes to proton secretion and luminal acidification. They also secrete HCO_3_^–^ through the CFTR channel. Lastly, basal cells transmit physiological cues, in particular during sexual arousal, which regulate the activity of principal and CCs.

When spermatogenesis is completed within the seminiferous tubules of the testis, spermatozoa are released in the lumen and collected through the rete testis and the efferent ducts to join the epididymal tract. The epididymis consists in a unique and highly convoluted tube of nearly 1 and 6 m long, in mice and humans, respectively (Hinton et al., 2011). It is anatomically divided into three principal regions, the caput, corpus, and cauda, following the proximal-distal axis (Hirsh, 1995; Turner, 2008); each of them being further divided in several segments as it is well-documented in rodents (10 and 19 segments in mouse and rats, respectively) (Johnston et al., 2007; Figure 2). In some species, specific morphological features can be observed, like in rodents, where a proximal segment adjoined to the testis, called the initial segment, is distinguishable (Soranzo et al., 1982). The epididymal epithelium is composed of different cell types, which are the principal cells (PCs), the clear cells (CCs), the narrow cells (NCs), the apical/basal cells and the halo cells together with immune cells such as macrophages and dendritic cells (Da Silva and Smith, 2015; Voisin et al., 2018; Breton et al., 2019; [Fig F2]). The proportion of each epididymal cell-type is variable between the different segments of the epididymis, which are associated with specific secretory and absorptive activities providing distinct luminal micro-environments along the tract contributing to sperm maturation (Breton et al., 2019). For instance, PCs, which are the most abundant cell type, are present in all epididymal regions while NCs exclusively locate to the initial segment and CCs are present in the caput and corpus, and highly enriched in the cauda (Hermo et al., 1992; Adamali and Hermo, 1996).

The epithelium of the epididymis constitutes a physical barrier which protects the sperm cells from the immune system and is essential to mediate vectorial transport of ions, solutes, nutrients and water from the blood circulation thus insuring sperm cell survival and protection (Hinton et al., 1995; Gregory and Cyr, 2014). Some of the maturation events occurring during sperm decent along the epididymal tract have been elucidated and it is now well-established that spermatozoa will acquire their motility when transiting through the caput and corpus regions before being stored until ejaculation, in a quiescent state in the caudal region (Bedford, 1967; Orgebin-Crist, 1967). Proteomic and transcriptional analysis of sperm retrieved from the different segments of the epididymis in several species, provided valuable information regarding the changes occurring during epididymal transit. Changes in surface protein content and post-translational modifications have been reported; sperm lipid composition is also described to be modified and influences membrane fluidity in preparation for egg fusion and fertilization (Gervasi and Visconti, 2017). Studies performed on the luminal fluid also highlighted variations in its composition along the epididymal tract, in respect to ions, soluble factors, proteins, non-coding RNA (Johnston et al., 2005; Dacheux et al., 2006, 2009; Yuan et al., 2006; Jelinsky et al., 2007; Dean et al., 2008; Moura et al., 2010; Guyonnet et al., 2011; Liu and Liu, 2015; Liu et al., 2015; Browne et al., 2019; for review see Sullivan and Mieusset, 2016; Zhou et al., 2018) most importantly, those studies uncovered the existence of small vesicles, called epididymosomes, which enable material transfer between epithelial and sperm cells and support their maturation, in the absence of intrinsic *de novo* transcription and translation events (Figure 2A; Sullivan et al., 2007; Frenette et al., 2010; for review see Sullivan et al., 2007; Zhou et al., 2018; Trigg et al., 2019).

### Epididymis Luminal Milieu, Ionic Fluxes, and pH

One important feature of epididymal maturation is the establishment of an acidic luminal fluid, which is required for sperm quiescence during their maturation and storage (Shum et al., 2011). Such specific luminal environment starts to be established within the efferent ductules, which exert an intensive reabsorption of the fluid released with spermatozoa from the testis (Clulow et al., 1998). The acidic pH of the epididymal luminal fluid is related to particular ionic composition, with low level of sodium, Cl^–^ and HCO_3_^–^ ions, in comparison to that of other organ fluids or blood plasma (Wales et al., 1966; Levine and Marsh, 1971; Jenkins et al., 1980). Overall, it is conferred by specific secretive and absorptive properties of each epithelial cell type and complex intercellular cross-talks ([Fig F2]). First, are involved the CCs, which are categorized as mitochondria-rich cells, and actively secrete protons via the V-ATPase proton pump, a multi-protein complex located at their apical side. In those cells, activation of the soluble adenylate cyclase (sAC) and a PKA-dependent pathway, trigger the accumulation of the V-ATPase pump at the plasma membrane from intracytoplasmic storage vesicles (Pastor-Soler et al., 2003; Belleannee et al., 2011; Battistone et al., 2018). The luminal ATP also stimulates membrane addressing of the V-ATPase pump in CCs, through pH-activated ATP purinergic membrane receptors such as P2 × 4 and elevation of the intracellular Ca^2+^ (Belleannee et al., 2011; Battistone et al., 2018). In addition, CCs also express the cytosolic carbonic anhydrase type II, which catalyzes hydration of carbon dioxide to HCO_3_^–^ and is therefore essential for acid/base transport (Breton, 2001). The PCs, which constitute the most abundant cell type of the epididymis are also very active in absorbing the HCO_3_^–^ in the proximal region of the mouse epididymis (initial segment) and in secreting protons through the sodium/hydrogen exchanger NHE3, in the distal region (Park et al., 2017; [Fig F2]). Last, the basal cells are also critical as they transmit physiological cues which regulate the activity of both principal and CCs (Leung et al., 2004; Cheung et al., 2005; Shum et al., 2008). In particular, during sexual arousal, prior to ejaculation, basal cells activate the secretion of HCO_3_^–^ by the PCs through the CFTR channel in a cAMP-PKA dependent manner (Park et al., 2017), an action which is hypothesized to prime the spermatozoa (Hagedorn et al., 2007; Pierucci-Alves et al., 2010; [Fig F2]). Interestingly, the luminal HCO_3_^–^ may also be incorporated into the CCs via the sodium HCO_3_^–^ co-transporter NBC (Jensen et al., 1999), and subsequently activate the sAC-PKA pathway triggering proton secretion. In this sense, CCs may behave as counteractors of luminal pH elevation, and be involved in the regulation of abnormal and/or sustained pH increase conditions. In addition, or alternatively, HCO_3_^–^ secretion by the PCs may be part of an integrated paracrine mechanism involving a crosstalk between clear and PCs, and ultimately leading to proton secretion by CCs and lumen acidification.

Overall, within the epididymal milieu, the established acidic pH and the low HCO_3_^–^ concentration need to be tightly regulated to insure proper sperm maturation and storage (for review see Bernardino et al., 2019). The importance of such epididymal intraluminal environment is well-demonstrated in various mouse models associated with abnormally elevated pH or ionic disequilibrium conditions, which all induce reduced sperm fertilization potential and male infertility (Yeung et al., 1998, 2004; Zhou et al., 2001; Bigalke et al., 2010; Weissgerber et al., 2012; for review see Zhou et al., 2018).

## Sperm Capacitation in the Female Genital Tract

### Definition, Function, and Associated Sperm Changes

Following ejaculation, spermatozoa achieve their ultimate functional maturation, which is initiated by secretions from the male accessory glands (prostate and seminal vesicles) and fully completed in the female genital tract, a process called capacitation. The process of capacitation was discovered by Chang (1951) and Austin (1952), respectively, who experimentally demonstrated that spermatozoa from rabbits and rats, need to spend enough time within the fallopian tubes of the female genital tract, in order to acquire their fertilization. These pioneer studies were immediately followed by investigations aiming at defining the minimal conditions permitting to capacitate sperm cells *in vitro* and the first successful experiment of *in vitro* fertilization was performed with hamster eggs by Yanagimachi and Chang (1963). This led to major achievements in reproductive medicine through the development of assisted reproduction technologies. Many additional investigations permitted to describe some of the molecular and biochemical events associated with capacitation, in humans and other species (see review Gervasi and Visconti, 2016), while sperm epididymal maturation was much less investigated and remains poorly understood.

**FIGURE 3:**
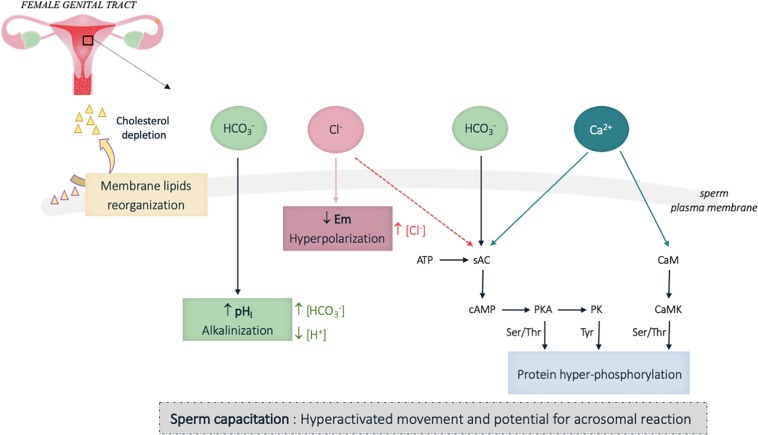
Schematic representation of the biochemical and electrophysiological changes during sperm capacitation in the female genital tract. Capacitation confers the sperm cells a hyperactivated motility characterized by an increased flagellar amplitude and beating frequency, and the ability to perform the acrosomal reaction and to specifically recognize and interact with oocyte. This functional activation is mainly induced by HCO_3_^–^, Ca^2+^ and Cl^–^ influxes, which trigger biochemical and electrophysiological changes in the cytoplasm and the whole plasma membrane of the sperm cells. Among the principal changes, are observed an increase of the plasma membrane fluidity through cholesterol depletion, which favor the relocation of proteins located in the sperm head and involved in oocyte interaction, together with an alkalinization of the cytoplasm and plasma membrane hyperpolarization. Intense protein phosphorylation, which include some proteins involved in flagellar beating, is observed on the sperm flagellum. pH_i_, intracellular pH; Em, membrane potential; […], cytoplasmic ion concentrations; ↑, increase; ↓, decrease. Plain and broken arrows indicate a direct and indirect effect, respectively.

It is now well-established that capacitation confers the sperm cells a hyperactivated motility characterized by increased flagellar amplitude and beating frequency, which enables sperm cells to penetrate through the cumulus cell layer surrounding the oocyte (Suarez, 2008). In addition, sperm acquire the ability to perform the acrosomal reaction and to specifically recognize and interact with oocyte (Tulsiani and Abou-Haila, 2011). This is associated with biochemical and electrophysiological changes occurring in the cytoplasm and the plasma membrane of the sperm cells, which together constitute some hallmarks of capacitation. Among these principal changes, were described an increase of the plasma membrane fluidity mainly due to cholesterol depletion, and complex ionic fluxes inducing alkalinization of the cytoplasm, plasma membrane hyperpolarization and flagellar protein phosphorylation (Figure 3; Visconti et al., 2011). HCO_3_^–^, Cl^–^, and Ca^2+^ ions were described to be involved in those processes. In particular, Ca^2+^ and HCO_3_^–^ directly bind to the soluble adenylate cyclase (sAC) and stimulate cAMP production (Chen et al., 2000; Jaiswal and Conti, 2003). The resulting increase in intracellular cAMP concentration is responsible for the activation of the protein kinase A (PKA) and subsequent phosphorylation cascades of flagellar proteins that are indispensable for sperm fertilization (Visconti et al., 2011; Figure 3). Among the phosphorylated targets, both axonemal and peri-axonemal proteins of the sperm flagellum were identified. Hence signaling proteins such as the AKAP proteins (Carrera et al., 1996) together with enzymes involved in energetic metabolism (pyruvate dehydrogenase and aldolase) (Arcelay et al., 2008) that locate to the fibrous sheath of the sperm flagellum are phosphorylated upon capacitation; other peri-axonemal components of the flagellum, such as the ODF (Mariappa et al., 2010) were also identified. Importantly, structural protein of the axoneme such as Tubulin (Arcelay et al., 2008) and dynein chains (Baker et al., 2010), which orchestrate flagellar beating (see Figure 1), are phosphorylated upon capacitation. Ca^2+^ also directly binds to CaM present in the sperm cells (head and flagellum) (Jones et al., 1980; Feinberg et al., 1981; Carrera et al., 1996) and regulates additional phosphorylation cascades initiated by the Calmodulin kinase (CaM kinase) (Gonzalez-Fernandez et al., 2012; Navarrete et al., 2015; see review Suarez, 2008). Overall, ion fluxes induced during capacitation tightly regulate both sperm flagellar beating and energy homeostasis.

### Sperm Capacitation, Ionic Fluxes, and pH

**FIGURE 4:**
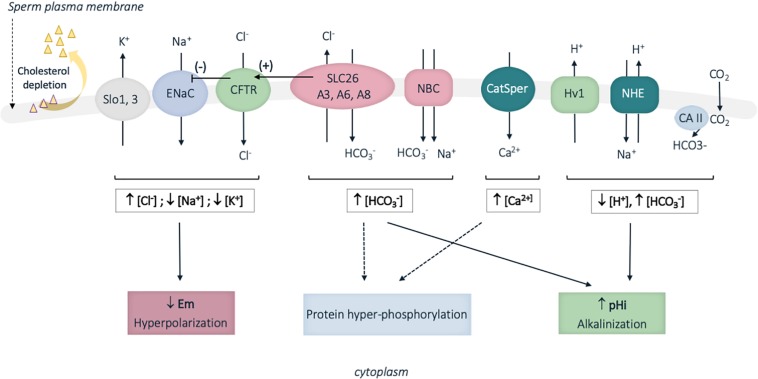
Schematic representation of some of the identified sperm membrane transporters involved in ion fluxes during human sperm capacitation. Cholesterol depletion occurring during capacitation also increases membrane fluidity. A panoply of ion transporters is involved in the complex ion fluxes, which induce membrane hyperpolarization, cytoplasm alkalinization and protein hyperphosphorylation. Slo13, sperm-specific K^+^ channel; ENaC, epithelial Na^+^ channel; CFTR, cystic fibrosis transmembrane conductance channel; SLC26, solute carrier 26; NBC, sodium HCO_3_^–^ transporter; CatSPER, sperm specific Ca^2+^ channel; Hv1, proton channel; NHE, Na^+^/H^+^ exchanger; CA, carbonic anhydrases; pH_i_, intracellular pH; Em, membrane potential; […], cytoplasmic ion concentrations; ↑, increase; ↓, decrease. Plain and broken arrows indicate a direct and indirect effect, respectively. CFTR stimulation by SLC26 proteins is represented by an arrow with (+) and ENaC inhibition by CFTR is represented by an arrow with (–).

Capacitation is induced by sperm exposition to high HCO_3_^–^ concentration and basic pH in the female genital tract, as compared to the acidic epididymal milieu (Zhou et al., 2005). Hence in contrast to the recorded HCO_3_^–^ concentrations of 2–7 nmol/L (pH 6.4; rats) in epididymal cauda (Levine and Marsh, 1971), sperm cells encounter HCO_3_^–^ concentrations of 30 nmol/L in the vas deferens (pH 7.5; rats) (Levine and Marsh, 1971), and approximatively 25 to 90 nmol/L in the fallopian tubes (pH 7.61; rabbit) (Vishwakarma, 1962; see review Ng et al., 2018). A panoply of ion transporters located at the surface of murine and human sperm cells was identified and shown to mediate some of the complex ionic fluxes during capacitation (Figure 4; Puga Molina et al., 2017). Briefly, the combined proton extrusion and HCO_3_^–^ influxes result in cytoplasm alkalinization. Proton extrusion from the sperm cells is mediated by the voltage-gated H^+^ channel (Hv1) in humans, and the Na^+^/H^+^ exchangers (NHE), also called SLC9 proteins, in humans, mice and rats (Puga Molina et al., 2017). Bicarbonate transporters involved in capacitation include members of the SLC4 sodium-dependent transporter and SLC26 Cl^–^/HCO_3_^–^ exchangers families (cf. infra), together with the CFTR Cl^–^ channel, which functions by cooperating with SLC26 Cl^–^/HCO_3_^–^ exchangers, in mouse and in humans (this later aspect regarding SLC26 proteins will be developed in the next section of the review). In addition, several isoforms of carbonic anhydrases (cytosolic and membrane) are present in the sperm and likely contribute in regulating sperm HCO_3_^–^ concentration (Puga Molina et al., 2017). The resulting cytoplasm alkalinization regulates pH-dependent channels; hence the Ksper and Slo outward potassium channels are activated (Navarro et al., 2007; Santi et al., 2010) while the inward sodium ENac channel is inhibited. This leads to hyperpolarization of the plasma membrane and in turn activates voltage- and pH-dependent channels. Among those, the CatSPER channel (cation channel sperm associated) is a multiprotein complex, which exclusively locates to the plasma membrane of the principal piece in human and mouse sperm flagellum, and mediates Ca^2+^ influxes (Figure 4; see review Singh and Rajender, 2015). In consistence with their restricted expression and function during capacitation, mutations in some genes encoding for some of the above ionic transporters (CatSPER1, CatSPER2, SLC26A3, and SLC26A8) were associated with male infertility due to asthenozoospermia, a pathology defined by reduced or absence of sperm motility (Hildebrand et al., 2010; Ray et al., 2017; Wedenoja et al., 2017).

## SLC26 Proteins in Sperm Function and Male Fertility

### Overview of SLC26 Protein Family

The Solute carrier 26 (SLC26) members are evolutionary conserved transmembrane proteins that mediate the transport of various anions including Cl^–^ (chloride), HCO_3_^–^ (bicarbonate), SO_4_^2–^ (sulfate), iodide (I^–^), formate (HCOO^–^) and C_2_O_4_^2–^ (oxalate), and contribute to the composition and the pH of secreted fluids in the body (Alper and Sharma, 2013). SLC26 belong to the highly conserved superfamily of amino acid-polyamine-organocation (APC) transporters and SLC26-related proteins are present in various organisms including bacteria, yeast, algae, plants (SulP/Sultr proteins) and non-mammalian vertebrates. In mammals, 10 members (SLC26A1 to SLC26A11; SLC26A10 being a pseudogene) have been identified, and are expressed throughout the body with organ-specific distribution (Alper and Sharma, 2013; Table 1). SLC26 proteins mainly function as secondary anion transporters (ion-coupled transporters), utilizing the electrochemical gradient of an ion to drive the transport of another solute against its gradient. Some of them also function as uncoupled electrogenic transporters similar to Cl^–^ channels (SLC26A7, A9) (Ohana et al., 2011; Alper and Sharma, 2013; Table 1). A few exception are to be mentioned: first, in mammals, no proper anion transport activity was reported for SLC26A5 (Prestin) (in contrast to chicken, zebra fish and insects) (Schaechinger and Oliver, 2007; Hirata et al., 2012) and SLC26A5 is supposed to act as a motor protein and to control outer hair cells of the cochlea in an anion-dependent manner (Zheng et al., 2000; Rybalchenko and Santos-Sacchi, 2008); second, the activity of SLC26A8 and SLC26A11, has been poorly investigated, and it is therefore difficult to precisely state on their anion specificity and mode of transport.

**TABLE 1:** Principal features of SLC26 family members.

**Protein**	**Major tissue distribution (mouse, human)**	**Anion transport features**	**Associated Human Diseases**	**Phenotype of the mouse models**	**References**
**SLC26A1 (SAT1)**	Kidney - Liver - Pancreas -Small intestine-Colon - Lung -Brain - Heart - Skeletal muscle - Male reproductive organs: mouse testis	SO_4_^2–^, Cl- and oxalate transporter	Nephrolithiasis	Deregulation of sulfate and oxalate homeostasis, nephrolithiasis. Defects of enamel maturation in mandibular incisors.	Lee et al., 2003; Regeer et al., 2003; Dawson et al., 2010, 2013; Gee et al., 2016; Yin et al., 2017
**SLC26A2 (DTDST)**	Ubiquitous with higher expression in the cartilage (chondrocytes) - Male Reproductive organs : human efferent ducts (ciliated cells)	SO_4_^2–^/2Cl^–^, SO_4_^2–^/2OH^–^, SO_4_^2–^/OH^–^/Cl^–^ exchanger	Growth retardation and Osteochondrodysplasia	Non-lethal chondrodysplasia	Hästbacka et al., 1994; Rossi et al., 1996; Haila et al., 2001; Forlino et al., 2005; Kujala et al., 2007; Ohana et al., 2012
**SLC26A3 (DRA/CLD)**	Gastrointestinal tract - Sweat Gland - Male reproductive organs: human testis (male germ cells), human efferent ducts (non ciliated cells), human epididymis ducts (apical mitochondrial rich cells), mouse spermatozoa	2Cl^–^/HCO_3_^–^, Cl^–^/OH^–^ exchanger NO_3_^–^ and SCN^–^ channel	Congenital Chloride Diarrhea. Human male subfertility	Chloride - losing diarrhea, Enhanced colonic proliferation. Loss of colonic fluid absorption and susceptibility to intestinal inflammation. Male infertility : epididymal defects, oligo-astheno-teratozoospermia, sperm capacitation defects	Silberg et al., 1995; Byeon et al., 1996; Hoglund et al., 1996; Melvin et al., 1999; Hihnala et al., 2006; Hoglund et al., 2006; Schweinfest et al., 2006; Shcheynikov et al., 2006; Dorwart et al., 2008; Chavez et al., 2012; Xiao et al., 2014; Wedenoja et al., 2017; El Khouri et al., 2018
**SLC26A4 (PDS/Pendrin)**	Thyroid - Inner ear - Kidney - Salivary gland duct	Cl^–^/HCO_3–_, Cl^–^/I^–^, I^–^/HCO_3_^–^ and formate exchanger	Pendred syndrome : deafness and goiter. Hypothyroidism	Deafness: enlargement of the membranous labyrinth and vestibular aqueduct, stria vascularis dysfunction. Impaired bicarbonate secretion in kidney. Acidic urine and hypercalciuria. Increased contractile force of aorta.	Li et al., 1998; Everett et al., 1999, 2001; Scott et al., 1999; Royaux et al., 2001, 2003; Amlal et al., 2010; Kim and Wangemann, 2010; Lu et al., 2011; Barone et al., 2012; Ito et al., 2014, 2015; Kopp, 2014; Sutliff et al., 2014; Mukherjee et al., 2017; Wen et al., 2019
**SLC25A5 (Prestin)**	Cochlea : outer hair cells	Antiporter: SO_4_^2–^/Cl^–^ and Cl^–^/HCO_3_^–^Motor protein: electromotility	Non syndromic deafness	Loss of outer hair cell electromotility, Loss of cochlear sensitivity	Zheng et al., 2000, 2003; Liberman et al., 2002; Liu et al., 2003; Cheatham et al., 2004; Muallem and Ashmore, 2006; Dallos et al., 2008; Mistrík et al., 2012
**SLC26A6 (CFEX/PAT1)**	Intestine - Stomach - Skeletal muscle - Heart - Kidney -Pancreas - Enamel - Male Reproductive organs : human efferent ducts (non ciliated cells), human ductus epididymis (apical mitochondria rich cells), mouse spermatozoa	Cl^–^/2HCO_3_^–^, Cl^–^/oxalate, Cl^–^/formate exchanger, NO_3_^–^ and SCN^–^ channel	Oxalate kidney stones; hyperoxaluria	Alteration of Cl-/HCO3- exchange in native pancreatic duct Calcium oxalate urolithiasis, Impairment of cardiac function, Oxalate secretion defects in saliva, Normal epididymal and sperm functions	Knauf et al., 2001; Jiang et al., 2002; Wang et al., 2002, 2005; Xie et al., 2002; Alvarez et al., 2004; Kujala et al., 2005; Freel et al., 2006; Jiang et al., 2006; Tuo et al., 2006; Ishiguro et al., 2007; Kujala et al., 2007; Monico et al., 2008; Seidler et al., 2008; Singh et al., 2010; Chavez et al., 2012; Song et al., 2012; Jalali et al., 2015; Sirish et al., 2017; Yin et al., 2017; El Khouri et al., 2018; Mukaibo et al., 2018
**SLC26A7 (SUT2)**	Kidney - Stomach - Inner ear cells - Male Reproductive organs : human testis and ductus epididymis (basal cells)	Cl^–^/HCO_3_^–^ exchanger Cl^–^ channel	Gastrointestinal dysfunction, Goitrous congenital hypothyroidism	Distal renal tubular acidosis, Impaired gastric acidification, Defects of enamel maturation in mandibular incisors, Normal hearing	Lohi et al., 2002; Petrovic et al., 2003; Petrovic et al., 2004; Kujala et al., 2005; Dudas et al., 2006; Kujala et al., 2007; Xu et al., 2009; Kim et al., 2014; Yin et al., 2017; Cangul et al., 2018; Ishii et al., 2019
**SLC26A8 (TAT1)**	Male Reproductive organs : human and mouse testis (male germ cells), spermatozoa	Cl^–^, SO_4_^2–^, oxalate transporter	Human male infertility (asthenozoospermia)	Male infertility, reduced sperm motility (asthenozoospermia), Normal epididymal function	Toure et al., 2001; Lohi et al., 2002; Kujala et al., 2007; Rode et al., 2012; Dirami et al., 2013; Touré, 2017
**SLC26A9**	Lung - Human bronchial epithelial cells - Stomach - Innear ear cells	Cl^–^/ HCO_3_^–^, Na+/anion exchanger Cl^–^ channel	Diffuse idiopathic bronchiectasis; Impaired exocrine pancreatic and lung functions in Cystic fibrosis patients	Impaired gastric secretion (gastric hypochlorhydria), Airway mucus obstruction in inflammatory condition, Reduction of renal chloride excretion, Elevated systemic arterial pressure, Impairment of intestinal electrolyte transport	Lohi et al., 2002; Xu et al., 2005;, Arcelay et al., 2008; Xu et al., 2008; Bertrand et al., 2009; Kim and Wangemann, 2010; Anagnostopoulou et al., 2012; Bakouh et al., 2013; Liu et al., 2015; Miller et al., 2015; Strug et al., 2016; Bertrand et al., 2017; Corvol et al., 2018
**SLC26A10 (Pseudogene)**	N/A	N/A	N/A	N/A	N/A
**SLC26A11 (SUT1/KBAT)**	Kidney, Intestine, Brain	SO_4_^2−^ transporter Cl^–^ channel	Renal tubular dysfunction, Impairment of locomotor coordination	Dysfunction of chloride homeostasis and neuronal activity in the cerebellum	Vincourt et al., 2003; Xu et al., 2011; Rahmati et al., 2013, 2016

*The table displays the tissues were SLC26 genes and proteins are mainly detected in humans and mice, together with their anion specificity and their mode of transport, when defined. The clinical signs of the human genetic diseases associated with SLC26 mutations and the phenotype of the corresponding mutant mouse models are also described. References for information summarized in the table are indicated. In blue are highlighted all the features in regard with male reproductive organs and this review.*

**FIGURE 5:**
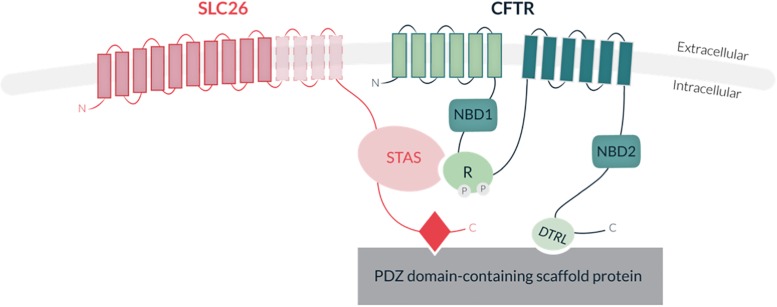
Schematic representation of SLC26 protein structure and interaction with the Cystic Fibrosis Transmembrane conductance Channel (CFTR). SLC26 proteins share a conserved transmembrane region of 10–14 hydrohobic spans, associated with their anion transport activity, and a cytoplasmic STAS domain (Sulfate Transporter and Anti-Sigma factor antagonist), involved in protein-protein interaction and regulation. Some members also contain a PDZ binding motif at their carboxy-terminal extremity. The CFTR protein consists of two transmembrane domains (TMD) (each containing six spans of alpha helices), two nucleotide-binding domains (NBD1 and NBD2) and a central regulatory domain (R-domain). CFTR activity is regulated by PKA-phosphorylation of the R-domain and ATP binding and hydrolysis at the two NBDs. Direct interaction of SLC26 with CFTR is mediated by the STAS domain and the regulatory (R) domain of CFTR. Indirect interaction of the proteins occurs through binding of both SLC26s and CFTR to common PDZ motif-containing scaffold proteins.

SLC26 proteins share a common structure, including a highly conserved transmembrane region with 10 to 14 spans, supporting the anion transport activity, and a cytoplasmic region, which comprises the STAS domain (Sulfate Transporter and Anti-Sigma factor antagonist) involved in SLC26 trafficking (Sharma et al., 2011; Bai et al., 2016), protein-protein interaction and regulation (for reviews see Ohana et al., 2011; Alper and Sharma, 2013; Figure 5). Several SLC26 members also carry a PDZ binding domain at their carboxy-terminal extremity (for reviews see Ohana et al., 2011; Alper and Sharma, 2013). Interestingly, most SLC26 members were shown to physically interact with the Cystic Fibrosis Transmembrane conductance Regulator channel (CFTR; MIM 602421) via their STAS domain, and to stimulate the CFTR Cl^–^ channel activity in various epithelia (Ko et al., 2004; Khouri and Toure, 2014; Figure 5). Such physical and functional cooperation highlights the cross-talks which are likely to exist between SLC26 and other ionic transporters. Interestingly, while SlC26 proteins were initially thought to function as monomers, some biochemical studies indicate that they can form homo- and hetero-dimers, providing an additional level of complexity in their mode of function and regulation (Chavez et al., 2012). Studies of the bacterial YeSLC26A2 protein indicated that homodimerization is supported by the transmembrane core and not by the cytoplasmic STAS domain (Compton et al., 2011) and recent work performed by Chang et al. (2019) achieved structural modeling of the membrane-embedded prokaryotic SLC26 dimer (SLC26Dg, *Deinococcus Geothermalis*. In mammals, such transmembrane homodimerization property was also described for SLC26A5 (Prestin) (Liu et al., 2003; Compton et al., 2011).

SLC26 proteins constitute one of the main classes of transporters that are involved in HCO_3_^–^ and pH homeostasis regulation; the other being SLC4, and HCO_3_^–^ transporters (see review Bernardino et al., 2019). In humans, their importance in maintaining correct ionic equilibrium and pH in various tissues and differentiation processes is demonstrated by the identification of SLC26 “loss of function” mutations in several hereditary genetic diseases: nephrolithiasis (SLC26A1), diastrophic dysplasia (SLC26A2), chloride loosing diarrhea (SLC26A3), Pendred syndrome -deafness and goiter- (SLC26A4), non-syndromic deafness (SLC26A5) and in men with reduced fertility and asthenozoospermia (SLC26A3, SLC26A8) (Everett and Green, 1999; Dawson and Markovich, 2005; El Khouri and Toure, 2014; Seidler and Nikolovska, 2019; Table 1). All the above phenotypes are in line with the nearly restricted tissue expression profiles observed for most of SLC26 genes. Notably, mutant mouse models have been generated for all SLC26 members, and all reproduced the clinical features of the SLC26 human-related diseases when applicable (Liberman et al., 2002; Forlino et al., 2005; Schweinfest et al., 2006; Touré et al., 2007; Dallos et al., 2008; Dror et al., 2010; El Khouri et al., 2018). In addition, studies of members not so far associated with human diseases (SLC26A7 and A11) revealed their functions in various tissues such as kidney, gastro intestinal tract, enamel, vestibular membrane of the cochlea, and brain (Xu et al., 2009; Rahmati et al., 2013, 2016; Kim et al., 2014; Yin et al., 2017), predicting that further investigations might lead to the identification of novel SLC26 gene mutations associated with pathophysiological conditions in humans (Table 1). Lastly SLC26 function may be critical for cystic fibrosis condition (CF; MIM 219700), a disease which is due to mutations in CFTR, and characterized by general defective electrolyte transport, chronic lung infections and inflammation, respiratory failure, digestive symptoms and male infertility (i.e., congenital bilateral absence of the vas deferens). Hence, genetic variants in SLC26A9, impairing the established cross-talk and interaction with CFTR contribute to the severity of respiratory and gastrointestinal symptoms observed in cystic fibrosis (see review El Khouri and Toure, 2014).

### SLC26 and CFTR Protein Functions in Sperm Cells and Epididymis

Among SLC26 proteins, SLC26A3, A6, and A8 were reported to locate to the human and mouse sperm and their functions were investigated through a range of cellular, biochemical and electrophysiological approaches (Chan and Sun, 2014; El Khouri and Toure, 2014). In addition, the generation and availability of knock out mouse models for all three proteins permitted to investigate *in vivo* their function and confirm some of the findings. Herein we will describe SLC26 protein functions in the sperm and epididymal cells, following their chronological order of discovery; in addition, as the CFTR channel was identified as a main interactor of SLC26 proteins, we will also describe the current knowledge about CFTR function in the sperm cells and male reproductive tract (Table 2).

**TABLE 2:** Principal characteristics and functions of SLC26A3, A6, A8, and CFTR in the sperm and epididymal cells.

**Protein**	**Expression in male reproductive organs**	**Protein location in sperm**	**Protein location in epididymis**	**Mouse KO phenotype in sperm**	**Mouse KO phenotype in epididymis**	**Mutations and associated diseases in Humans**	**References**
**SLC26A3 (DRA/CLD)**	Human: testis (male germ cells), efferent ducts (non-ciliated cells) and ductus epididymis (apical mitochondrial rich cells. Mouse: spermatozoa	Flagellum: midpiece	Luminal border of apical mitochondrial rich cells	Homozygous Slc26a3-null mice: Oligo-astheno-terato-zoospermia, Sperm capacitation defects	Homozygous Slc26a3 knock-out mice: Epididymal dysplasia and granulome in the cauda	Homozygous loss of function: Congenital Chloride Diarrhea (CLD) with male subfertility (oligo-astheno-zoospermia) Heterozygous loss of function: Male infertility (asthenozoospermia)	Hihnala et al., 2006; Hoglund et al., 2006; Chavez et al., 2012; Wedenoja et al., 2017; El Khouri et al., 2018
**SLC26A6 (CFEX/PAT1)**	Human: efferent ducts (non-ciliated cells), ductus epididymis (apical mitochondrial rich cells). Mouse: spermatozoa	Flagellum: midpiece	Apical mitochondrial rich cells	Homozygous Slc26a6-null mice: No sperm phenotype	Homozygous Slc26a6 knock-out mice: No epididymal phenotype	Not reported	Kujala et al., 2007; El Khouri et al., 2018
**SLC26A8 (TAT1)**	Human and mouse: testis (male germ cells), spermatozoa.	Flagellum : annulus Equatorial segment	Head: Not expressed	Homozygous Slc26a8-null mice: Asthenozoospermia Sperm capacitation defects	Homozygous Slc26a8 knock-out mice: No epididymal phenotype	Heterozygous loss of function: Male infertility (asthenozoospermia)	Toure et al., 2001; Lohi et al., 2002; Kujala et al., 2007; Hildebrand et al., 2010; Rode et al., 2012; Dirami et al., 2013
**CFTR**	Human: efferent ducts (non-ciliated cells), ductus epididymis (apical mitochondrial rich cells), vas deferens spermatozoa. Mouse: spermatozoa	Flagellum: midpiece. Equatorial segment	Head: Luminal border of apical mitochondrial rich cells	Heterozygous CFTR tm1Unc: reduced sperm capacitation and fertilization potential	Homozygous DeltaF508 (DeltaF/DeltaF) and knock-out (cf/cf) CFTR mice: normal epididymis but collased lumen of the vas deferens	Homozygous loss of function: Cystic fibrosis (CF); Isolated obstructive azoospermia in non-CF patients : congenital bilateral absence of the vas deferens, (CBAVD)	Tizzano et al., 1994; Reynaert et al., 2000; Patrizio and Salameh, 1998; Claustres, 2005; Hernández-González et al., 2007; Touré et al., 2007; Xu et al., 2007 (Book chapter: https://doi.org/10.1159/000477279)

*The table summarizes the tissues within the male reproductive organs where SLC26A3, A6, A8, and CFTR genes are expressed and the detection of the corresponding proteins in sperm and epididymal cells. The sperm and epididymal phenotypes in the mouse knock out mouse models are reported together with the phenotype associated with mutations in those genes in humans. References for information summarized in the table are indicated.*

### SLC26A8 Function in Sperm Cells

SLC26A8 (also called Testis Anion transporter 1) was cloned almost concomitantly by Touré et al. in 2001 (Toure et al., 2001) and Lohi et al. (2002), and reported to be exclusively expressed in human testis and in the male germ cells (Toure et al., 2001; Lohi et al., 2002). It was shown to interact with MgcRacGAP, a regulator of small Rho GTPases, later identified to be required for cytokinesis, in somatic and germ cells (Maddox and Oegema, 2003; Lores et al., 2014). SLC26A8 was the first member to be investigated in male reproductive functions as the remarkable tissue-specificity suggested that it might fulfill critical function in the sperm cells. As mentioned above, the anion transport activity of SLC26A8 has been poorly investigated. First studies from Touré et al. (2001) indicated a Cl^–^-dependent SO_4_^2–^ transport when the protein was expressed in COS cells, suggesting that SLC26A8 might function as a coupled ion transporter. To date the activity of SLC26A8 toward the HCO_3_^–^, which physiological relevance in sperm cell function is established, has not been reported. Ubiquitous invalidation of *Slc26a8* gene was performed in the mouse by homologous recombination, and resulted in male sterility due to total sperm immotility while viability was unaffected. Functional analysis of *Slc26a8*-null sperm indicated reduced ATP consumption and the absence of capacitation-associated protein phosphorylation (Touré et al., 2007). SLC26A8-null sperm also displayed structural defects of the annulus, which induced a hairpin bending of the flagellum (Touré et al., 2007). In line with this phenotype, the SLC26A8 protein was found to locate at the annulus and equatorial segment of mouse and human sperm (Touré et al., 2007; Lhuillier et al., 2008; Rode et al., 2012).

### CFTR Function in Sperm Cells

In the same time, publications from different laboratories indicated the expression and functions of the CFTR channel during sperm capacitation. In addition to its expression in the respiratory, digestive and genital epithelia, the CFTR channel was shown to be expressed in mature sperm from mice, guinea pigs and humans where it contributes to Cl^–^ and HCO_3_^–^ fluxes during capacitation (see review Touré, 2017). Hence in 2007, Xu and coworkers identified the CFTR channel at the equatorial segment of the sperm head in both mouse and human sperm. They showed that sperm treatment with a selective inhibitor of CFTR (CFTRinh-172), prevents the increases in intracellular cAMP, and pH and the membrane hyperpolarization, which are required for proper capacitation and acrosome reaction. The study of a heterozygous mouse model for CF (CFTRtm1Unc) also indicated low fertilization capacity, with impaired sperm motility and capacitation (Xu et al., 2007). Hernández-González et al. (2007) concomitantly reported the expression of CFTR in mouse and human sperm but found it to be restricted to the midpiece of the flagella; they showed that CFTR is required for membrane potential hyperpolarization during capacitation by regulating the epithelial sodium channels (ENaC). Those findings were categorically confirmed by work from Figueiras-Fierro et al. (2013) who specified CFTR currents in mouse sperm cells by means of patch clamp measurements on wild-type vs. CFTR ΔF508-null sperm and by using specific CFTR agonists and antagonists. More recently Puga Molina et al. (2017) also demonstrated that in human sperm, CFTR activity is required for capacitation-associated phosphorylation in a PKA-dependent manner.

### SLC26A8 and CFTR Cooperation in Sperm Cells

Following the discovery of CFTR protein and activity in mature sperm, Rode et al. (2012) demonstrated that SLC26A8 interacts with the CFTR channel via the STAS domain; the molecular complex was also identified by immunoprecipitation on mouse testis protein extracts. By means of radioactive iodide efflux measurements in CHO-K1 cells and patch clamp experiments in Xenopus oocytes, they demonstrated that SLC26A8 stimulates the CFTR Cl^–^ transport activity (Rode et al., 2012). They further studied the Slc26a8 knock out model and demonstrated that the absence of capacitation-associated protein phosphorylation and motility could be partially rescued when supplementing with cAMP permeant analogs. They also demonstrated that the soluble adenylate cyclase (sAC) relocates properly at the annulus of Slc26a8-null sperm, despite their structural defects. This indicated that overall, the motility and capacitation defects observed in SLC26A8-null sperm result from a functional deregulation; therefore *in fine* SLC26A8 localization at the annulus may be conciliated with the regulation of anion fluxes (Rode et al., 2012). Overall these data suggested that SLC26A8 actively and/or indirectly contribute to Cl^–^ and HCO_3_^–^ influxes required for the activation of c-AMP-dependent protein phosphorylation during capacitation.

### SLC26A3 Function in Sperm Cells

SLC26A3, also called Down Regulated in Adenoma (DRA), was cloned in 1993 by Schweinfest et al. (1993) and later showed to encode for an intestinal anion transport molecule (Silberg et al., 1995) whose mutations lead to congenital Chloride Loosing Diarrhea (CLD; [MIM 214700]) (Hoglund et al., 1996), an autosomal recessive disorder due to defective intestinal electrolyte absorption (Kere et al., 1999; Wedenoja et al., 2011). SLC26A3 acts as a Cl^–^/HCO_3_^–^ exchanger (PMID 10428871) with a 1:1 or 2:1 stoichiometry (see review Seidler and Nikolovska, 2019) and is principally expressed in the enterocytes of the gastrointestinal tract epithelium, in humans and rodents (Jacob et al., 2002), where it is responsible for high HCO_3_^–^ output rates in the mid to distal region of the colon (Xiao et al., 2012, 2014). Chen et al. (2009) reported for the first time the expression of SLC26A3 in sperm cells from guinea pig. They showed that the protein locates to the equatorial segment and colocalizes with the CFTR channel. Importantly they demonstrated that Cl^–^ was required for the HCO_3_^–^-dependent changes occurring during capacitation (pH and cAMP rise, protein phosphorylation); they proposed a cooperative model involving SLC26A3 for HCO_3_^–^ entry (in exchange of Cl^–^ efflux) and CFTR for Cl^–^ recycling pathway. In 2012, Chavez et al. (2012) reported the expression of SLC26A3 transcripts in mouse spermatogenic cells (spermatocytes, spermatids) and found SLC26A3 protein to be restricted to the sperm flagellum midpiece, similar to the CFTR channel. Chavez et al. (2012) also conducted a series of *in vitro* measurements on mouse epididymal sperm, using MQAE and DISC_3_, two fluorescent probes reflecting the intracellular Cl^–^ content and membrane hyperpolarization, respectively. These assays were performed in presence or absence of a set of anion transport antagonists: the CFTRinh172, which specifically targets CFTR, or Tdap and 5099, which presumably target SLC26A3 (to date, their specificity and selectivity among SLC26 members were not proven). From those data, Chavez et al. (2012) concluded that both SLC26A3 and CFTR are involved in regulating the Cl^–^ influx and HCO_3_^–^-induced hyperpolarization upon capacitation.

### SLC26A6 Function in Sperm Cells

In the course of their study, Chavez et al. (2012) similarly analyzed SLC26A6, also called PAT-1, CFEX. SLC26A6 is highly expressed in pancreas, kidney (Lohi et al., 2000; Waldegger et al., 2001) and in the intestine (Wang et al., 2002) where it functions as a Cl^–^/HCO_3_^–^ exchanger, both in mouse and in humans; although the stoichiometry seems to differ between the two species as an electroneutral exchange was measured in human cells it is electrogenic in the mouse (Cl^–^/HCO_3_^–^, 1:2) (Seidler and Nikolovska, 2019). Chavez et al. (2012) demonstrated that in sperm cells, SLC26A6 co-localized with SLC26A3 and CFTR to the sperm flagellum midpiece. They also demonstrated by co-immunoprecipitation that, *in vivo*, SLC26A6 is part of the SLC26A3/CFTR protein complex. However, *in vitro*, the use of DOG and PMA, two compounds that are described to inhibit SLC26A6 through PKC activation, did not alter Cl^–^ influx nor membrane hyperpolarization upon capacitation, suggesting that SLC26A6, in contrast to SLC26A3, is not critical for those processes (Chavez et al., 2012). Taken together the above studies indicate that in the sperm cells, the SLC26A3 and A8 proteins locate to the flagellum midpiece and annulus, respectively, and cooperate with the Cl^–^ CFTR channel in regulating Cl^–^ influx, membrane hyperpolarization and protein phosphorylation during capacitation. In addition, SLC26A3, A8 and CFTR are likely to colocalized at the equatorial segment of mouse and human sperm and to control acrosomal reaction. In mouse, although shown to locate to the sperm cells, the SLC26A6 protein seems dispensable for capacitation.

### SLC26A3 Function in Epididymal Cells

Last year, El Khouri et al. (2018) investigated *in vivo*, the function of SLC26A3 and SLC26A6 proteins, in sperm functionality by analyzing ubiquitous knock out mouse models previously generated to study their functions in the gastrointestinal system (Singh et al., 2010; Xiao et al., 2012). They showed that in addition to the previously reported phenotype of congenital diarrhea (Xiao et al., 2012; Singh et al., 2013), SLC26A3−null mice displayed severe lesions and abnormal cytoarchitecture of the cauda epididymis, which strongly impacted the reserve of sperm cells in epididymides (El Khouri et al., 2018). This phenotype is in line with the subfertility previously observed for a few CLD affected men (Hoglund et al., 2006). SLC26A3−null mice showed a drastic reduction of the cauda size with reduced tube sections observed within the epididymis while the caput regions appeared overall not affected; in addition, the number of the presence of granuloma and fibrosis was observed, indicative of an inflammatory context and a disruption of the blood−epididymal barrier. The limited number of sperm cells, which was produced, failed to swim and was not responsive to the induction of capacitation as protein phosphorylation could not be obtained when supplementing with HCO_3_^–^ and Ca^2+^. Similar to what was observed for SLC26A8-null sperm, a partial rescue of protein hyperphosphorylation was obtained when adding cAMP permeant analog, indicating a failure to activate the soluble adenylate cyclase. In addition, Slc26a3-null sperm exhibited abnormal morphology with increased proportion of bent and coiled flagellum, and the proportion of reacted acrosome was also significantly increased. Importantly, similar analyses were performed in parallel, on SLC26A6 knock out mice and no epididymal, nor spermatic defects were observed. This indicated that although co-expressed with SLC26A3 in sperm and epididymal cells, *in vivo*, SLC26A6 is not critical for sperm production and functionality. This could be due to subtle differences in anion transport activity as different stoichiometry and ion specificity were reported for these two members (i.e., A3 and A6); in addition, their activities could also vary between the different tissues where they are expressed, due to tissue-specific partners/regulators.

In humans, SLC26A3, SLC26A6, and CFTR proteins were detected on the luminal border of the apical mitochondria-rich cells (AMRC) of the ductus epididymis (Hihnala et al., 2006; Kujala et al., 2007) while SLC26A8 was absent (Kujala et al., 2007). In the mouse, El Khouri et al. (2018) detected SLC26A3 and SLC26A6 transcripts in epididymes while SLC26A8 was absent. Ruan et al. (2012) detected CFTR protein in the PCs of the mouse cauda epididymis while it was absent in other epithelial cell types. CFTR protein was aslo recently detected on the apical membrane of mouse caput epididymis and in the smooth muscle myoid cells (Sharma and Hanukoglu, 2019). Overall, such expression patterns are in line with the observed epididymal phenotype in SLC26A3 knock out mice and suggests an impairment of electrolyte homeostasis, which may impact the pH and ionic content of the epididymal milieu and prevent sperm maturation. Supporting this hypothesis, an increased amount of V ATPase protein was observed in SLC26A3−null epididymis caput compared to wild type tissues, a compensatory mechanism reflecting HCO3- and pH deregulation within the luminal fluid (Shum et al., 2009, 2011; Breton et al., 2016). As observed in the context of the sperm cells, although present within the epididymal ductus, SLC26A6 function seems to be dispensable for epididymal cytoarchitecture and functionality. Lastly, SLC26A8 protein was found absent from the epididymal tract (Kujala et al., 2007; Wedenoja et al., 2017; El Khouri et al., 2018) and rationally no epididymal phenotype was observed in the knock-out mice (Touré et al., 2007).

Taken together those studies indicate that among the three SLC26 proteins investigated in the male reproductive organs, only SLC26A3 appears critical for epididymal luminal milieu and sperm maturation. SLC26A3 is likely to contribute to the cellular cross-talks regulating HCO_3_^–^ fluxes and leading to luminal acidification. SLC26A3 function in epididymal electrolyte regulation probably relies on interaction and/or cooperation with CFTR channel, and the Na^+^/H^+^ antiporter 3, NHE3, which is expressed in both the intestine and in the male reproductive system (Melvin et al., 1999; Lamprecht et al., 2002; Hihnala et al., 2006). Hence Wang et al. (2017) recently reported that NHE3−deficient mice display ultrastructural defects of the epididymis and the vas deferens, as well as significant reduction of CFTR protein levels in these structures, ultimately leading to male infertility. Such phenotype is comparable to that of Slc26a3-null mice and support the hypothesis of a multi−channel protein complex, CFTR/SLC26A3/NHE3, involved in electrolyte regulation and luminal acidification of the epididymal ductus.

### SLC26 Dysfunctions in Human Male Infertility

Following the identification of SLC26A8, genetic investigations in human infertility were promptly initiated owing to its exclusive expression in the testis and the male germ cells. Hence in 2005, Mäkelä et al. screened a cohort of 83 men with oligo- and azoospermia but did not identified variants in SLC26A8 genes associated to this phenotype (Makela et al., 2005). In 2013, based on the phenotype of Slc26a8-null mice, Dirami et al. (2013) screened a cohort of 146 men consulting for infertility and displaying moderate asthenozoospermia. Asthenozoospermia is defined by a reduction or an absence of sperm motility (less that 32% of progressive sperm, following the values established by the World Health Organization) (Cooper et al., 2010) and is found in nearly 80% of infertile men (Curi et al., 2003). Dirami and collaborators identified three heterozygous missense variants, c.260G > A (p.Arg87Gln), c.2434G > A (p.Glu812Lys), and c.2860C > T (p.Arg954Cys), which they showed to be absent from control individuals and to impact the functional cooperation between SLC26A8 and CFTR. They demonstrated, *in vitro*, that while physical interaction was not altered, all identified variants conducted to reduced protein amounts of SLC26A8 and that of the associated CFTR channel. They showed that *in vitro*, SLC26A8 protein amounts could be restored to control levels by proteasome inhibition, indicating that the variants impacted protein stability, likely by inducing deleterious protein conformational changes (Dirami et al., 2013). These three mutations were identified at the heterozygous state, in contrast to mutations identified in other SLC26-related diseases, which all segregate following an autosomal recessive mode (Dawson and Markovich, 2005); this could be attributed to the fact that only men displaying asthenozoospermia of moderate severity were screened in this study.

Following a similar strategy, Wedenoja et al. published in 2017, the screening of a cohort of 283 asthenozoospermic men and the identification of the c.2062 G > C (p.Asp688His) heterozygous variant in SLC26A3, in 3.2% of the patients (Wedenoja et al., 2017). Analysis of the variant’s frequency in Exac database indicated that it is enriched in the Finnish population. Furthermore, functional studies showed that the p.Asp688His variant did not impact SLC26A3 intrinsic Cl^–^/HCO_3_^–^ exchange activity, nor its protein amount. The p.Asp688His variant, which locates to the STAS domain, was able to interact with the CFTR channel but failed to stimulate CFTR Cl^–^ transport activity, *in vitro*, in Xenopus oocytes. Here again, the variant was identified at the heterozygous level, but in this case, this might be consistent with the fact that *SLC26A3* homozygous loss of function induce Chloride Loosing Diarrhea and subfertility, a much more severe phenotype (Wedenoja et al., 2011).

## Discussion

SLC26 constitute one of the largest family of membrane proteins but their functions have only been recently investigated, mainly in the gastrointestinal and renal tissues where they appear critical for electrolyte transport and pH regulation. The male and female genital tracts also rely on pH homeostasis but few investigations were performed in the reproductive organs. As described in this review, recent work was performed by means of electrophysiological and *in vitro* studies on human and mouse sperm, which permitted to describe the critical role of SLC26A3 and A8 in regulating the electrophysiological and biochemical changes occurring in the sperm cells during capacitation. In addition, the phenotypical characterization of Slc26 knock-out mouse models together with translational studies of human infertility conditions, permitted to confirm their physiological relevance for sperm fertilization potential; importantly, the requirement of SLC26A3 for proper peididymal structure and functions was uncovered.

One of the main research gaps to be overtaken concerns the delineation of each SLC26 member contribution, in those processes. In this regard, a comprehensive and comparative analysis of SLC26 cellular and subcellular expression patterns, within the male reproductive tract, is cruelly missing. Hence, except SLC26A8, for which a clear-cut expression pattern is available (i.e., exclusively sperm specific), the expression pattern of SLC26 members is still unclear and the observed protein localizations, as in the case of SLC26A3, were found to diverge between different laboratories. The development of high-throughput sequencing technologies and single cell analyses has facilitated the access to large public expression datasets and therefore constitutes one asset to further progress in this field. In particular, few expression databases dedicated to the reproductive tissues have been established: The ReproGenomics viewer (Darde et al., 2015, 2019) and The Mammalian Reproductive Genetics database. When analyzing three distinct RNAseq datasets from mouse purified germ cells, conflicting results were obtained as compared to the assumed expression pattern of SLC26 members in the sperm cells. Hence while SLC26A6 and A8 transcripts were clearly detected in the mouse germ cells from spermatogonia to spermatid stage (Gan et al., 2013; Green et al., 2018; Lukassen et al., 2018), SLC26A3 transcripts were not detected at all. The analyses of the same datasets also exclude any expression of SLC26A1, A4, A5, and A9 in the mouse germ cells while SLC26A2, A7, and A11 were detected. Similar analyses of epididymal expression dataset, through the Mammalian Reproductive Genetics database, indicated that SLC26A3 transcripts are readily detected in the mouse epididymis, which is coherent with transcript and protein detection in mouse and in human epididymides that were reported by distinct laboratories, (Hihnala et al., 2006; El Khouri et al., 2018).

The discrepancy regarding SLC26A3 expression in the germline really question the presence and the function of SLC26A3, if any, in the mouse sperm cells. These conflicting data result from the limited biochemical tools available to analyze SLC26 protein expression, and potentially from antibody cross-reaction with different SLC26 members. Importantly, the situation is very different in humans as analysis of RNAseq data indicated that SLC26A3 transcripts are detected in human differentiating germ cells, as opposed to the mouse germ cells. This clearly alerts about the risk of generalizing data obtained from one specie to another. The differences in cytoarchitecture and compartmentations of the epididymis ductus between humans and mouse together with the difference in the sperm “status” used in studies (epididymal sperm in mouse vs. ejaculated sperm in human) constitute additional arguments, if required, to prohibit data transfer from one specie to another.

Considering the profound epididymal defects reported in the Slc26a3 knock out mouse model, an important connected point is to determine whether the dysfunctions observed in Slc26a3-null sperm when performing *in vitro* capacitation, could result from defects initially occurring during epididymal maturation. Hence it is highly probable that functional defects occurring within the epididymis could impair sperm “priming” and later prevent proper response to capacitation in the female genital tract. This point is particularly important to address, considering the uncertain expression of SLC26A3 in the sperm cells. The discrimination between those two intricated processes (epididymal priming and maturation vs. capacitation in the female genital tract) constitutes a requisite if one wants to better define sperm post-testicular maturation events; in the future this may be performed by generating conditional mutant mouse models with gene invalidation restricted to the epididymal epithelium or to the germ cell lineage.

Lastly, an important progress concerns the development of pharmacological compounds specifically targeting SLC26 proteins. To date some compounds such as Tenidap, 5099, DOG and PMA, were utilized, *in vitro*, to inhibit SLC26 protein functions in the sperm cells but their inhibitory mechanisms are sometimes indirect or unknown; in addition, no information is available regarding their selectivity among SLC26 members, which may limit the interpretation of the data and even lead to confusion in specifying the contribution of each member. A major work in this field was recently published by Haggie et al. (2018) who identified SLC26A3 specific antagonists with the aim of treating gastrointestinal defects. The authors performed a fluorescent high-throughput screening based on the Cl^–^/I^–^ exchange activity mediated by SLC26A3 and a halide-sensitive yellow fluorescent probe (YFP). The strength of this study relies on the demonstrated selectivity of the identified compounds by means of various and complementary approaches: *in vitro* study on different representative members of the SLC26 family (human and mouse orthologs) together with *in vivo* studies using a mouse model with gastrointestinal defects resulting from SLC26A3 dysfunction. In the future, the use of such compounds will definitely help to better understand SLC26 functions and molecular mechanisms in the processes of sperm post-testicular maturation and fertilization potential. The study of other SLC26 members expressed in the epididymal and sperm cells, such as SLC26A2, A7 and A11, will also provide additional information regarding the multiple cross-talks and hierarchical regulatory mechanisms between SLC26 and other ion transporters.

## Author Contributions

AT performed the request bibliography analyses and wrote the manuscript.

## Conflict of Interest

The author declares that the research was conducted in the absence of any commercial or financial relationships that could be construed as a potential conflict of interest.
